# Shear wave elastography of the diaphragm in acute exacerbation of chronic obstructive pulmonary disease: A prospective observational study

**DOI:** 10.1097/MD.0000000000033329

**Published:** 2023-03-17

**Authors:** Jingfeng Zhang, Chunfeng Zhang, Lijuan Yan, Lei Zhang, Yanping Wan, Qi Wang, Peng Wang, Jinzhi Xu

**Affiliations:** a Department of Ultrasound, Baoji High-Tech Hospital, Shaanxi, China; b Department of Respiratory and Critical Care Medicine, Baoji High-Tech Hospital, Shaanxi, China; c Department of Ultrasound, Baoji Central Hospital, Shaanxi, China; d Department of Radiology, the First Affiliated Hospital of Xi’an Jiaotong University, Shaanxi, China; e Department of Radiology, Baoji High-Tech Hospital, Shaanxi, China.

**Keywords:** acute exacerbation of chronic obstructive pulmonary disease, diaphragmatic, shear wave elastography, stiffening, ultrasound

## Abstract

Patients with acute exacerbation of chronic obstructive pulmonary disease (AECOPD) are prone to diaphragmatic dysfunction. However, dynamic assessment of diaphragmatic function is complex and difficult, and whether the assessment of diaphragmatic function can reflect clinical indicators such as lung function in AECOPD patients remains unclear. We studied diaphragm stiffness and diaphragm stiffening rate (DSR) in AECOPD patients with acute exacerbations ≥ 2 times within 1 year and their correlation with clinical data, the diaphragmatic thickening fraction (DTF), lung function, and blood gas values.

In total, 112 AECOPD patients in group C and Group D who had acute exacerbations ≥ 2 times within 1 year in the Global Initiative for Chronic Obstructive Lung Disease Guideline A (low risk, few symptoms), B (low risk, many symptoms), C (High risk, few symptoms), D (High risk, many symptoms) grouping system were included in the study. Their general clinical data, chronic obstructive pulmonary disease assessment test (CAT), modified medical research council (mMRC), number of acute exacerbations in 1 year, DTF, lung function, and blood gas analysis were collected. The diaphragm shear wave elasticity at functional residual capacity (DsweFRC) and DSR were measured by ultrasound.

The DsweFRC and DSR of Group D were higher than those of Group C (*P* < .05). DsweFRC, DSR were negatively correlated with DTF, forced expiratory volume in 1 second (FEV1), forced vital capacity (FVC) and FEV1/FVC (*r* ranged from −0.293 to −0.697, all *P* < .05), and positively correlated with CAT score, mMRC score, and arterial carbon dioxide pressure (*r* ranged from 0.274 to 0.462, all *P* < .05) in both groups; the correlation coefficients of DsweFRC, DSR and DTF, FEV1/FVC in group D were greater than those in group C. There was no correlation between DsweFRC, DSR and arterial oxygen partial pressure in both groups (*P* > .05). The DsweFRC, DSR increased with the number of acute exacerbations per year in both groups.

We found that diaphragmatic stiffness in AECOPD patients increased with the number of acute exacerbations within 1 year, correlated with DTF, CAT, mMRC, lung function, and arterial carbon dioxide pressure and provides a simple and practical method for dynamically assessing diaphragmatic function and disease severity in AECOPD patients.

## 1. Introduction

According to the World Health Organization, chronic obstructive pulmonary disease (COPD) is the third leading cause of death worldwide after coronary heart disease and stroke.^[1]^ The diaphragmatic dysfunction in COPD patients is caused by airway obstruction, lung hyperdistension due to increased respiratory resistance, decreased protein synthesis, malnutrition, increased muscle apoptosis, and other factors.^[2–4]^ The diaphragmatic dysfunction affects respiratory function and ultimately increases the risk of respiratory failure and mortality.^[5]^ Antenom et al^[6]^ found that the diaphragm function of patients with acute exacerbation of chronic obstructive pulmonary disease (AECOPD) decreased continuously with the development of the disease. Therefore, dynamic evaluation of diaphragm function in AECOPD patients is very important.

The transdiaphragmatic pressure is the Global Initiative for Chronic Obstructive Lung Disease (GOLD) standard for evaluating diaphragmatic function, which is the pressure difference between the esophagus and the stomach cavity. However, it is difficult to perform it on a large scale in clinical practice, because the measurement of transdiaphragmatic pressure is invasive and has technical limitations.^[7]^ Ultrasound measurement of the diaphragm thickening fraction (DTF) is a feasible method to evaluate diaphragm function in COPD patients.^[8]^ Because there is a strong linear relationship between muscle shear modulus and muscle strength,^[9,10]^ muscle strength and muscle function can be evaluated by ultrasonic shear wave elastography (SWE) measurement of the muscle tissue shear modulus. Therefore, ultrasound SWE is an alternative method to evaluate the diaphragm function.

The severity evaluation of COPD patients includes lung function and quality of life. The committee of the Global Initiative for Chronic Obstructive Pulmonary Disease points out that the classification of COPD patients into groups A, B, C, and D based on their chronic obstructive pulmonary disease assessment test (CAT), modified medical research council (mMRC score) and history of acute exacerbations can guide the evaluation of disease severity, disease course, and efficacy.^[11]^ The International Primary Care Respiratory Group published a “User Guide for COPD Health Tools,” which ranked various health status questionnaires in terms of validity and reliability,^[12]^ pointed out that the CAT was the preferred questionnaire. Several studies have found that diaphragmatic dysfunction is associated with pulmonary function in patients with stable COPD.^[13–15]^ In a prospective clinical trial conducted by Xu et al^[16]^, it was found that ultrasonic SWE could quantitatively assess the diaphragmatic stiffness in patients with stable COPD, and it was found that the diaphragmatic stiffness in patients with stable COPD was correlated with the CAT score and pulmonary function. However, it is not clear whether diaphragmatic stiffness changes with the progression of AECOPD or whether diaphragmatic stiffness is correlated with pulmonary function, CAT, mMRC score or other clinical data during acute exacerbation.

In this study, diaphragm shear wave elasticity at functional residual capacity (DsweFRC) was considered the diaphragm stiffness. The primary objective of our study was to detect the characteristics of DsweFRC and diaphragm stiffening rate (DSR) in C and D groups of AECOPD patients; the secondary objective was to detect the correlation of DsweFRC and DSR with clinical characteristics such as pulmonary function and blood gas analysis. This study provides a new method for the real-time dynamic assessment of diaphragm function and disease severity in AECOPD patients.

## 2. Materials and methods

### 2.1. Design and participants

This prospective study was approved by the Ethics Committee of Baoji High-tech Hospital (No. 2022-002), and each participant signed informed consent. Patients with AECOPD admitted to the Department of Respiratory and Critical Care Medicine of Baoji High-tech Hospital from October 2021 to October 2022 were recruited. The COPD patients were divided into groups A, B, C, and D according to CAT score, mMRC score and the number of acute exacerbations within a year using the GOLD guideline A (low risk, few symptoms), B (low risk, many symptoms), C (High risk, few symptoms), D (High risk, many symptoms) grouping system, as shown in Table 1. A total of 112 COPD patients with acute exacerbations ≥ 2 times within 1 year were enrolled, including 87 males and 25 females. They were divided into 2 groups: group C (n = 58) and group D (n = 54).

**Table 1:** The 2020 GOLD guidelines for COPD assessment form.

Group	Features	Number of acute exacerbations within 1 yr	CAT	mMRC
A	Low risk, few symptoms	≤1	<10	0–1
B	Low risk, many symptoms	≤1	≥10	≥2
C	High risk, few symptoms	≥2	<10	0–1
D	High risk, many symptoms	≥2	≥10	≥2

CAT = chronic obstructive pulmonary disease assessment test, COPD = chronic obstructive pulmonary disease, mMRC = modified medical research council.

The study inclusion criteria were as follows: confirmed AECOPD patients who met the criteria of the GOLD diagnosis and had an acute exacerbation; the patient was conscious and could complete the CAT and mMRC questionnaires; each AECOPD patient could cooperate with the pulmonary function test and diaphragm ultrasound examination; and the patient’s forced expiratory volume in 1 second (FEV1)/forced vital capacity (FVC) was <70% after the application of a bronchodilator. The exclusion criteria were as follows: severe heart, liver and renal organ dysfunction, pneumonia and bacterial infection in other organs, bronchial asthma, severe bronchiectasis, digestive tract diseases, active pulmonary tuberculosis, malignant tumors, and other consumptive diseases. Patients with disorders such as anxiety, depression and cognitive dysfunction were excluded for accuracy. The clinical data collected included age, sex, body mass index, systolic blood pressure, diastolic blood pressure, white blood cell count, C-reactive protein, CAT score, mMRC score, number of acute exacerbations within 1 year, FEV1, FVC, FEV1/FVC, arterial oxygen partial pressure (PaO_2_), and arterial carbon dioxide pressure (PaCO_2_).

### 2.2. Ultrasonic measurement

DTF and diaphragm SWE were measured using a GE S8 ultrasonic system and a 9 L linear array transducer (9 MHz). All ultrasound examinations were performed by a physician with more than 5 years of ultrasound experience.

**Figure 1.**
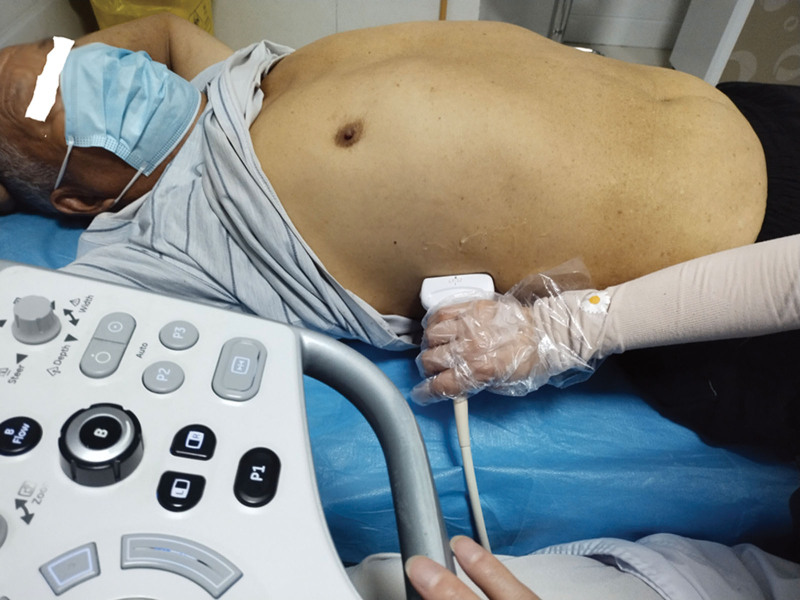
Examination position of the patient and position of the ultrasound probe.

**Figure 2.**
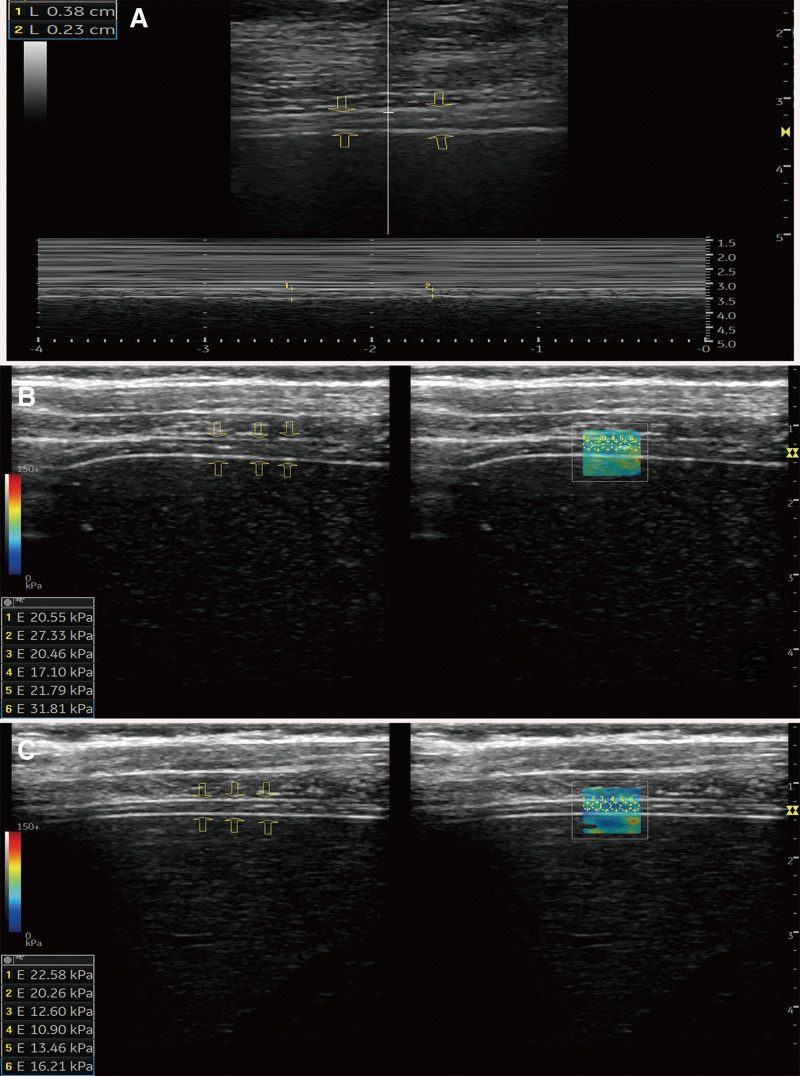
Ultrasound images of diaphragm in a 58-year-old man with AECOPD. (A) The end-inspiratory and end-expiratory diaphragmatic thickness was 0.38cm and 0.23mm, respectively, and the calculated diaphragm thickening fraction was 0.65. (B) The average diaphragm shear wave elasticity at total lung capacity was 23.17KPa in the sampling frame. (C) The average diaphragm shear wave elasticity at functional residual capacity was 16.00 KPa in the sampling frame. After calculation, diaphragm stiffening rate was 0.45. AECOPD = acute exacerbation of chronic obstructive pulmonary disease.

For DTF measurement, the patient was in the supine position, and the transducer was placed vertically in the 8th to 10th intercostal area of the right anterior axillary line (see Fig 1). Ultrasound showed that the diaphragm was a 3-layer structure, with 2 hyperechoic lines representing the peritoneum and pleura and a hypoechoic middle layer representing the diaphragm, which thickened during inspiration and thinned during exhalation. The patient was asked to breathe normally, and the diaphragm was clearly visualized. After freezing the image, the diaphragm thickness was measured 3 times at the end of inspiration and the average value was taken as the end-inspiratory diaphragm thickness; the diaphragm thickness was measured 3 times at the end of expiration and the average value was taken as the end-expiratory diaphragm thickness; diaphragm thickening fraction = (end-inspiratory diaphragm thickness - end-expiratory diaphragm thickness)/end-expiratory diaphragm thickness. The diaphragmatic thickness was measured 3 times both at the end of inspiration and at end of expiration, and the average value was taken as the final end-inspiration and end-expiration diaphragmatic thickness. DTF = (end-inspiratory diaphragm thickness - end-expiratory diaphragm thickness)/end-expiratory diaphragm thickness.^[17]^ (see Fig. 2A).

For diaphragmatic SWE measurement, the patient was supine with the right arm above the neck, and the transducer was placed at the right 8th to 10th intercostal and anterior axillary areas (see Fig 1). The transducer was used to span 2 rib spaces to avoid compression. To maintain the same position of the probe for repeated measurements, a marker was painted on the chest of the patient. After the diaphragm was clearly displayed, the SWE sampling frame was placed at the diaphragm, diaphragmatic SWE maps were acquired at total lung capacity and functional residual capacity, respectively. Six circular regions of interest were overlaid on the sampling frame of the SWE maps to obtain the elastic modulus of shear waves in the 6 regions of interests, which were summed and averaged as the diaphragmatic shear wave elasticity of total lung capacity and DsweFRC.^[16]^ DSR = (diaphragmatic shear wave elasticity of total lung capacity - DsweFRC)/DsweFRC.^[18]^ (see Fig. 2B and C).

The DsweFRC of the first 15 patients was examined by the original examiner and another physician with 3 years of ultrasound experience to test the interexaminer reliability. On the second and third days after the first examination of the first 15 patients, the original examiner performed the second and third examinations of DsweFRC to test the internal reliability of the same examiner.

### 2.3. Statistical analysis

This was an observational study using ultrasound SWE to measure diaphragm stiffness, so we referred to previous studies, and a sample size of 50 patients per group was sufficient.^[16]^ SPSS 24.0 statistical software (IBM Corporation, Armonk, NY) was used to analyze and process all data. The Kolmogorov–Smirnov method was used to test the normality of the measurement data. Variables with a normal distribution were expressed as the mean ± SD, comparisons between the groups were analyzed by the t test, and the correlation between 2 variables was analyzed by Pearson correlation analysis. Nonnormally distributed data were presented as medians (*Q*1, *Q*3), comparisons between the groups were performed using the nonparametric Wilcoxon rank-sum test, and correlations between variables were analyzed by Spearman correlation analysis. The chi-square test was used for comparison of enumeration data. Interclass correlation coefficients (ICC) were used to assess the intra-assessor and inter-assessor reliability of the DsweFRC measures. When *P* < .05, the difference was statistically significant. A scatter plot was drawn using GraphPad Prism 5.

## 3. Results

The general clinical data of groups C and D are shown in Table 2. Table 3 shows that the number of acute exacerbations differed between groups C and D within 1 year (*P =* .007).

**Table 2:** Clinical information of group C and D.

Characteristic	Group C (N = 58)	Group D (N = 54)	*P* value
Age, yr	72.2 ± 10.4	70.7 ± 11.2	.462
Male, n (%)	49 (84%)	38 (70%)	.073
BMI, kg/m^2^	22.3 ± 3.0	22.7 ± 2.2	.373
SBP (mm Hg)	121.0 ± 15.9	118.8 ± 17.9	.545
DBP (mm Hg)	72.7 ± 8.5	74.3 ± 9.8	.318
White blood cell (×10^9^)	10.1 ± 3.9	11.8 ± 3.1	.785
C-reactive protein (mmol/L)	68.0 (10.5169.6)	78.8 (11.5180.0)	.677
FEV1 (mL)	1158.0 (825.0, 1988.5)	814.9 (667.7,1739.8)	.006
FVC (mL)	1985.0 (1352.1,2478.5)	1749.3 (1276.0,2280.0)	.023
FEV1/FVC (%)	45.9 ± 11.5	38.83 ± 10.8	.001
PaO_2_ (mm Hg)	67.3 (52.7,84.5)	62.4 (44.1,88.3)	.019
PaCO_2_ (mm Hg)	45.2 (42.5, 69.0)	61.1 (50.0, 81.9)	.001
CAT score	8.1 ± 1.5	18.1 ± 1.9	.000
mMRC score	1.1 ± 0.4	2.5 ± 0.6	.000
DTF	0.30 (0.21,0.50)	0.21 (0.15,0.40)	.043

Continuous variables are presented as the median (interquartile range) and mean ± SD. Nominal variables are presented as total number (percent).

CAT = chronic obstructive pulmonary disease assessment test, DTF = diaphragmatic thickening fraction, mMRC = modified medical research council, FEV1 = forced expiratory volume in 1 second, FVC = forced vital capacity, PaCO2 = arterial carbon dioxide pressure, PaO2 = arterial oxygen partial pressure.

**Table 3:** Comparison of the number of acute exacerbations within 1 year between the 2 groups.

	Number of cases	2 times	3 times	≥4 times
Group C	58	23	30	5
Group D	54	13	24	17
*P* value	.007			

The internal reliability of the values measured by the same examiner at different times was good (ICC = 0.789, *P* = .000, 95% CI 0.584–0.915), and the external reliability of the variables measured by the different examiners was also good (ICC = 0.727, *P =* .001, 95% CI 0.355–0.900).

**Figure 3.**
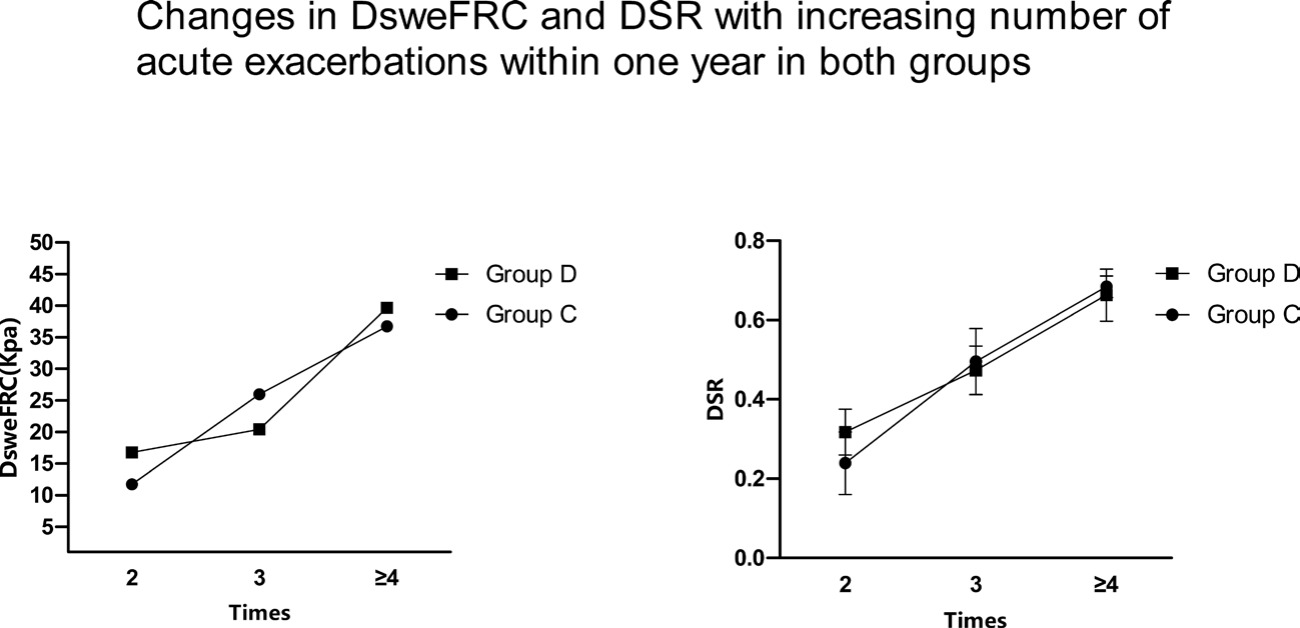
shows that DsweFRC and DSR in groups C and D increased with the number of acute exacerbations within 1 year. DsweFRC = diaphragm shear wave elasticity at functional residual capacity, DSR = diaphragm stiffening rate.

Table 4 shows that the DsweFRC and DSR of the AECOPD patients in the 2 groups and the DsweFRC and DSR in group D were significantly higher than those in group C (all *P* < .05). There was a tendency for DsweFRC and DSR to increase with the number of acute exacerbations within 1 year in both groups. (See Fig. 3).

**Table 4:** Comparison of DsweFRC and DSR between groups C and D.

Parameter	Group C (n = 35)	Group D (n = 33)	*P* value
DsweFRC	18.52 (12.2, 28.5)	20.58 (18.08, 39.0)	.040
DSR	0.41 ± 0.17	0.50 ± 0.14	.004

Continuous variables are presented as the median (interquartile range) and mean ± SD.

DSR = diaphragm stiffening rate, DsweFRC = diaphragm shear wave elasticity at functional residual capacity.

In group C, DsweFRC showed low negative correlation with DTF, FEV1/FVC, FVC (*r* ranged from −0.273 to −0.492, all *P* < .05), and weak negative correlation with FEV1 (*r* = −0.293, *P* = .026); low positive correlation with CAT score, PaCO_2_ (*r* ranged from 0.306–0.309, all *P* < .05), weak positive correlation with mMRC score (*R* = 0.274, *P* = .037), and no correlation with PaO_2_(*R* = 0.057, *P* = .670); DSR showed low negative correlation with DTF, FEV1/FVC, FEV1, FVC (*r* ranged from −0.308 to −0.430, all *P* < .05), and low positive correlation with CAT score, mMRC score and PaCO_2_ (*r* ranged from 0.303–0.398, all *P* < .05) had a low positive correlation, and no correlation with PaO_2_(*r* = −0.030, *P* = .823). (see Table 5) In group D, DsweFRC, DSR all showed moderate negative correlation with DTF, FEV1/FVC (*r* ranged from −0.538 to −0.697, all *P* = .000), and low negative correlation with FEV1 and FVC (*r* ranged from −0.304 to −0.386, all *P* < .05); low positive correlation with CAT score, mMRC score, PaCO_2_ (r ranged from 0.302–0.462, all *P* < .05), and no correlation with PaO_2_(all *P* > .05). (see Table 6).

**Table 5:** Correlation of DsweFRC and DSR with clinical characteristics such as pulmonary function and blood gas analysis in group C.

Variables	DsweFRC		DSR	
	*r*	*P* value	*r*	*P* value
DTF	−0.492	.000	−0.430	.001
FEV1/FVC	−0.408	.001	−0.429	.001
FEV1	−0.293	.026	−0.308	.018
FVC	−0.373	.004	−0.308	.018
CAT score	0.306	.021	0.398	.002
mMRC score	0.274	.037	0.303	.021
PaCO_2_	0.309	.018	0.308	.019
PaO_2_	0.057	.670	−0.030	.823

CAT = chronic obstructive pulmonary disease assessment test, DSR = diaphragm stiffening rate, DsweFRC = diaphragm shear wave elasticity at functional residual capacity, DTF = diaphragmatic thickening fraction, FEV1 = forced expiratory volume in 1 second, FVC = forced vital capacity, mMRC = modified medical research council, PaCO2 = arterial carbon dioxide pressure, PaO2 = arterial oxygen partial pressure.

**Table 6:** Correlation of DsweFRC and DSR with clinical characteristics such as pulmonary function and blood gas analysis in group D.

Variables	DsweFRC		DSR	
	*r*	*P* value	*r*	*P* value
DTF	−0.697	.000	−0.670	.000
FEV1/FVC	−0.538	.000	−0.623	.000
FEV1	−0.306	.024	−0.304	.026
FVC	−0.373	.005	−0.386	.004
CAT score	0.302	.027	0.395	.003
mMRC score	0.349	.010	0.462	.000
PaCO_2_	0.328	.015	0.310	.023
PaO_2_	0.082	.556	−0.074	.597

CAT = chronic obstructive pulmonary disease assessment test, DSR = diaphragm stiffening rate, DsweFRC = diaphragm shear wave elasticity at functional residual capacity, DTF = diaphragmatic thickening fraction, FEV1 = forced expiratory volume in 1 second, FVC = forced vital capacity, mMRC = modified medical research council, PaCO2 = arterial carbon dioxide pressure, PaO2 = arterial oxygen partial pressure.

In group D, DsweFRC, DSR all showed moderate negative correlation with DTF, FEV1/FVC (*r* ranged from −0.538 to −0.697, all *P* = .000), and low negative correlation with FEV1 and FVC (*r* ranged from −0.304 to −0.386, all *P* < .05); low positive correlation with CAT score, mMRC score, PaCO_2_ (r ranged from 0.302–0.462, all *P* < .05), and no correlation with PaO_2_ (all *P* > .05).(see Table 6).

## 4. Discussion

COPD patients have stiffened diaphragms. The diaphragmatic muscle fibers mainly contain type I and type II muscle fibers. The type I muscle fibers resist fatigue, and the type II muscle fibers generate strength.^[19]^ COPD patients develop disease, which ultimately causes diaphragmatic muscle fiber refactoring because of chronic hypoxia and hypercapnia in the diaphragm, imbalance in protein synthesis and degradation, increases in local inflammation, and oxidative stress, endocrine disorders, disuse atrophy, reduced capillary density in skeletal muscle, malnutrition, drugs (e.g., glucocorticoids) and many changes in the systems of the local microenvironment.^[2]^ Other studies have found that the length of the diaphragm is related to the degree of lung distension,^[20]^ and the higher the ratio of gas volume and residual volume to the total lung volume, the shorter the length of muscle. All the above factors make the diaphragm muscle fiber adaptively change, eventually leading to greater metabolic capacity and resistance to fatigue.^[20,21]^ Therefore, in COPD patients, the number of diaphragm type I muscle fibers increase, and the number of type II fiber muscle fibers decrease. Testelmans D^[22]^ and Barreiro E^[23]^ found that COPD patients had more type I fibers than type II muscle fibers in the diaphragm than healthy people. Several studies have found that type I muscle fibers are stiffer than type II fibers.^[24–26]^ Xu et al^[16]^ found that severe COPD patients had stiffer diaphragms than patients with mild-to-moderate COPD. In this study, we found that the DsweFRC and DSR in group D was significantly higher than that in group C (*P* < .05), which confirmed that the stiffness of the diaphragm increased with the aggravation of the disease. After further study, we also found that the DSR of AECOPD patients also increased with aggravation of the disease. There has been no report on DSR in previous studies.

Elnemr,^[27]^ and Eryuksel et al^[28]^ reported that the measurement of the diaphragm thickening rate by ultrasound can accurately and quickly evaluate the diaphragm function. Brown et al^[29]^ found that the mitochondrial density of type I muscle fibers in the diaphragm was higher than that of type II muscle fibers. With the exacerbation of AECOPD, the diaphragm muscle strength decreases, DTF decreases, type I muscle fibers increase, diaphragm mitochondrial density increases, and fiber density increases. According to the shear modulus calculation formula, the higher the tissue density, the higher the shear modulus.^[30]^

The CAT scores included cough, phlegm, chest tightness, feeling of going up a flight of stairs or climbing a hill, housework activities, going out, confidence, and sleep and energy, which reflected the severity of the symptoms. The CAT scores increased, the symptoms worsened, the type I fibers increased in the diaphragm, and DsweFRC and DSR increased. Paulin et al^[8]^ found that with the exacerbation of AECOPD, dyspnea increased, the mMRC score increased, the diaphragmatic activity decreased, and the diaphragmatic function decreased. Therefore, with the increase in the CAT and mMRC scores in AECOPD patients, DsweFRC and DSR also increase.

Some common indicators of airway obstruction include FEV1/FVC%, FEV1, and FVC. The higher the above lung function indexes, the better the pulmonary ventilation capacity; the lower the value, the more serious the airflow obstruction. Scheibe et al^[3]^ found that the airway obstruction in COPD patients increases the workload of the respiratory muscles, especially the diaphragm, resulting in diaphragm fatigue and reduced diaphragm function. With the exacerbation of AECOPD, the degree of airflow obstruction increases, FEV1/FVC%, FEV1, FVC decrease, diaphragm function further decreases, type I fibers increase, diaphragm mitochondrial density increases, and diaphragm stiffness and DSR increase. Therefore, DsweFRC and DSR increase with decreasing FEV1/FVC%, FEV1 and FVC in AECOPD patients.

There was no correlation between PaO_2_ and DsweFRC and DSR, suggesting that the PaO_2_ index could not directly reflect the diaphragm function and airway obstruction in patients with AECOPD. PaCO_2_ was positively correlated with DSR and DsweFRC, which may be related to decreased diaphragm function, reduced CO_2_ output and significantly higher partial pressure of CO2 in patients with AECOPD.^[31]^ The decrease in diaphragm function is accompanied by an increase in diaphragm type I fibers and an increase in mitochondrial density, which leads to an increase in diaphragm stiffness.

Our study also has some limitations. First, the patients in Group A and Group B in the GOLD A (low risk, few symptoms), B (low risk, many symptoms), C (High risk, few symptoms), D (High risk, many symptoms) grouping system had a low risk of acute exacerbation, so we did not include them in the study, which may bias the results of the study. Second, the ultrasound imaging of the left diaphragm was not ideal due to gastric gas interference, and we only studied the stiffness of the right diaphragm. Third, we only studied the diaphragm stiffness at functional residual capacity, and the characteristics of the diaphragm stiffness at total lung capacity are not clear and need to be further studied.

## 5. Conclusion

Ultrasound SWE measurement of diaphragmatic stiffness in AECOPD patients can evaluate diaphragmatic function in different risk groups, evaluate disease progression, and provide a basis for individualized treatment of AECOPD patients. Therefore, it is a good prospect for clinical application.

## Author contributions

**Conceptualization:** Jingfeng Zhang, Chunfeng Zhang.

**Data curation:** Yanping Wan, Jinzhi Xu.

**Funding acquisition:** Jingfeng Zhang.

**Formal analysis:** Chunfeng Zhang.

**Investigation:** Chunfeng Zhang, Lei Zhang.

**Methodology:** Jingfeng Zhang, Peng Wang.

**Project administration:** Jingfeng Zhang, Chunfeng Zhang.

**Resources:** Lijuan Yan, Peng Wang.

**Supervision:** Qi Wang.

**Writing – original draft:** Jingfeng Zhang, Yanping Wan.

**Writing – review & editing:** Lijuan Yan, Lei Zhang.
